# Clinical Application of Wearable Devices in Stroke Rehabilitation: A Scoping Review of Safety, Feasibility, and Adherence

**DOI:** 10.3390/healthcare14152400

**Published:** 2026-08-05

**Authors:** Shuchang Xu, Xunna Yin, Shanshan Tian, Yatong Zhang, Peijie Wang, Yangang Zhao, Wei Liu

**Affiliations:** 1 School of Nursing, Shandong University of Traditional Chinese Medicine, Jinan 250355, China; 2024110457@sdutcm.edu.cn (S.X.); zhangyatong2023@126.com (Y.Z.); 13031748770@163.com (P.W.); 2 Guang’anmen Hospital Jinan Branch, Jinan 250000, China; 15634558979@163.com (X.Y.); 15165055640@163.com (S.T.); 3 School of Traditional Chinese Medicine, Shandong University of Traditional Chinese Medicine, Jinan 250355, China

**Keywords:** stroke, wearable devices, rehabilitation, safety, feasibility, adherence

## Abstract

**Highlights:**

**What are the main findings?**

- Wearable devices are generally safe for stroke rehabilitation, with most adverse events being mild and manageable.
- Patient adherence is high in clinical settings but more variable in home-based rehabilitation due to device burden and self-management challenges.

**What are the implications of the main findings?**

- Wearable devices can enable personalized and high-intensity rehabilitation, highlighting the importance of user-friendly and comfortable designs.
- Successful transition from clinical to home-based rehabilitation requires integrating remote monitoring and structured guidance to optimize adherence and safety.

**Abstract:**

Background/Objectives: Stroke is a major cause of long-term disability worldwide. Wearable devices have shown potential in supporting rehabilitation, yet evidence on their safety, feasibility, and adherence remains fragmented. This scoping review aimed to evaluate the safety, feasibility, and adherence of wearable devices in stroke rehabilitation. Methods: A systematic search of PubMed, EMBASE, Web of Science, and CINAHL Complete was conducted in January 2026. Studies involving participants with a confirmed diagnosis of stroke were included regardless of study design. Studies were eligible if wearable devices were used as part of rehabilitation interventions and reported outcomes related to safety, feasibility, or adherence. Adverse events were categorized according to severity, while feasibility was assessed using indicators such as recruitment rate, dropout rate, and adherence rate. Results: A total of 38 studies involving 1121 participants were included in this review. Available evidence generally suggested a favorable safety profile for wearable device-assisted rehabilitation, with most reported adverse events being mild and primarily consisting of skin irritation, pressure-related discomfort, muscle fatigue, localized pain, and transient discomfort. Recruitment rates ranged from 5.8% to 100%, indicating substantial variability in implementation feasibility across studies. Only four studies reported adherence-related outcomes, and considerable variation existed in the definitions and assessment methods used. Most included studies reported intervention completion, attendance, or dropout outcomes rather than directly evaluating adherence. Conclusions: Current evidence suggests that wearable devices are generally safe and potentially feasible for stroke rehabilitation. However, substantial heterogeneity across studies, inconsistent adverse event reporting, and limited adherence data restrict the strength of current conclusions. Adherence remains an important evidence gap, as it was infrequently reported and inconsistently defined. Future research should establish standardized reporting criteria for safety, feasibility, and adherence outcomes and evaluate wearable device interventions in larger and more diverse populations with longer follow-up periods.

## 1. Introduction

Stroke is one of the leading causes of death and long-term disability worldwide [1]. Global Burden of Disease studies have shown that approximately 11.9 million new stroke cases occurred worldwide in 2021, and around 93.8 million people were living with the consequences of stroke. Stroke remains the third leading cause of death and disability globally [2,3]. In addition, the number of disability-adjusted life years (DALYs) attributable to stroke has continued to rise over recent decades, highlighting the persistent public health challenge posed by this condition [3,4].

Following a stroke, motor dysfunction is the primary cause of disability in patients [5]. The restoration of motor function is a central component of stroke rehabilitation [6,7]. Early and regular exercise training plays a significant role in promoting neural plasticity, improving functional recovery, and reducing disability rates [8,9,10]. The underlying mechanisms of neural recovery may partly involve intracellular signaling cascades, including the Ras/Raf/MEK/ERK pathway, which has been implicated in neuronal survival and plasticity following ischemic injury [11]. Therefore, exercise rehabilitation is crucial for stroke patients.

Conventional rehabilitation therapy typically relies on face-to-face guidance from physical therapists and involves repetitive task-oriented training, functional exercises, and gait training to facilitate neuroplasticity [12,13]. However, in real-world clinical settings, the intensity and duration of rehabilitation training are often limited by factors such as healthcare resources, therapist availability, and patient adherence. In addition, many patients have difficulty maintaining structured rehabilitation programs after hospital discharge, which may negatively affect long-term recovery outcomes [6,9]. Exploring innovative technological approaches that can support long-term training and enhance rehabilitation intensity has become an important research focus.

In recent years, wearable technologies have rapidly developed in the field of rehabilitation medicine. Wearable devices typically integrate sensors, actuators, or stimulation systems with the human body to provide functions such as movement assistance, activity monitoring, biofeedback, and neuromodulation [14]. For example, exoskeletons or robotic assistive systems can provide mechanical support to improve gait or upper limb movement, while systems based on inertial sensors can monitor movement patterns and provide real-time feedback to optimize training performance [14,15]. Compared with traditional rehabilitation approaches, wearable devices offer advantages such as portability, continuous monitoring capability, and the potential to support home-based rehabilitation, which has attracted increasing research interest in stroke rehabilitation [16].

In rehabilitation research, safety generally refers to the occurrence of intervention- or device-related adverse events during the implementation of an intervention, including falls, skin injuries, exacerbation of pain, injuries caused by device malfunction, and other adverse reactions [17]. Feasibility reflects the practicality of implementing an intervention in a target population and specific setting and is commonly assessed through indicators such as recruitment and retention rates, device acceptability, technical reliability, training completion rates, and resource requirements [18]. Adherence refers to the extent to which participants continuously use a device or complete training according to the prescribed protocol and is typically evaluated using measures such as device wearing time, training completion rate, and frequency of use [19].

Although previous studies suggest that wearable devices may have potential benefits in promoting motor recovery after stroke, the existing evidence remains heterogeneous in terms of device types, intervention protocols, study designs, and application settings. Moreover, evidence regarding the safety, feasibility, and patient adherence associated with these devices remains scattered. The characteristics of wearable device use in clinical versus home-based environments also lack systematic synthesis. To date, no scoping review has systematically synthesized evidence across the three critical dimensions of safety, feasibility, and adherence for wearable devices in stroke rehabilitation, nor has the comparative evidence across clinical and home-based settings been comprehensively mapped. This gap underscores the need for a unified synthesis to guide future research and clinical translation. Therefore, a comprehensive mapping of the current evidence is necessary to better understand the role of wearable technologies in stroke rehabilitation and to identify future research directions.

Although wearable devices have been investigated in stroke rehabilitation for many years, recent advances in flexible sensors, intelligent exoskeletons, the Internet of Things (IoT), and artificial intelligence (AI) have substantially improved device integration, data acquisition capabilities, and home-based rehabilitation applications. Consequently, the functionality and application modes of wearable technologies have evolved considerably in recent years [20]. Considering the rapid pace of technological development, studies published after 2020 are more likely to reflect the current state of wearable technologies and their clinical applications in stroke rehabilitation. Therefore, this review focuses on studies published between 2020 and 2026 to provide the most up-to-date evidence with practical relevance.

Accordingly, this review systematically summarizes the current research on wearable devices in stroke rehabilitation published between 2020 and 2026; categorizes the characteristics of different device types and application scenarios; and synthesizes evidence regarding their safety, feasibility, and adherence. By identifying existing research gaps, this review aims to inform future clinical implementation and technological development.

## 2. Materials and Methods

This scoping review was conducted in accordance with the Preferred Reporting Items for Systematic Reviews and Meta-Analyses extension for Scoping Reviews (PRISMA-ScR) guidelines [21]. The scope of this review was defined using the Population–Concept–Context (PCC) framework. The population consisted of adults diagnosed with stroke. The concept focused on the use of wearable devices in stroke rehabilitation, particularly their safety, feasibility, and adherence. The context referred to rehabilitation interventions conducted in clinical, community, or home-based rehabilitation settings [21,22]. This scoping review was retrospectively registered on the Open Science Framework (OSF) (https://doi.org/10.17605/OSF.IO/JS4EN).

### 2.1. Identifying the Research Question

This review aimed to address the following research questions:

(a) What evidence exists regarding the safety, feasibility, and adherence of wearable devices used in stroke rehabilitation?
(b) What types of wearable devices are currently used in stroke rehabilitation, and in which rehabilitation settings (clinical, community, or home-based) are they applied?
(c) What characteristics of participants are reported in studies using wearable devices for stroke rehabilitation?

### 2.2. Inclusion/Exclusion Criteria

#### 2.2.1. Inclusion Criteria

Studies were included if they met the following criteria:

- Participants were adults with a confirmed diagnosis of stroke.
- Wearable devices were used as part of rehabilitation interventions or supportive tools during motor rehabilitation.
- The device was worn on the body (e.g., wrist, trunk, upper or lower limbs).
- The study reported at least one outcome related to safety, feasibility, or adherence, such as adverse events, recruitment rate, dropout rate, adherence rate, or acceptability.
- The study was conducted in rehabilitation settings, including clinical rehabilitation centers, community settings, or home-based rehabilitation.
- The study was an original research article with extractable data.
- Articles were published in peer-reviewed journals between 2020 and 2026.
- Publications were available in English or Chinese.

#### 2.2.2. Exclusion Criteria

- Conference abstracts, educational articles, book chapters, reviews, or other non–peer-reviewed literature.
- Studies focusing solely on the measurement properties of sensors or the association between sensor data and clinical assessment outcomes.
- Studies centered on large fixed equipment, robotic systems, or gaming consoles rather than wearable devices.

### 2.3. Databases and Systematic Search

We conducted a systematic search of PubMed, Embase, Web of Science, and CINAHL in January 2026. Search terms included stroke, stroke survivors, wearable devices, rehabilitation, safety, feasibility, adherence, and adverse events, together with their related synonyms and controlled vocabulary terms. Because indexing systems and subject headings differ across databases, database-specific search strategies were developed and adapted accordingly while maintaining consistency in the core search concepts. The complete search strings for all databases, including Boolean operators and subject headings, are provided in File S1. In addition, the reference lists of all included studies were manually screened to identify potentially relevant studies not captured through database searching.

Given the rapid development of wearable rehabilitation technologies in recent years, including flexible sensors, intelligent exoskeletons, Internet-of-Things-enabled systems, and artificial intelligence-assisted devices, the characteristics and application scenarios of contemporary wearable technologies differ substantially from those investigated in earlier studies. To reflect the current state of technological development and clinical implementation, the search was restricted to studies published between January 2020 and January 2026.

### 2.4. Screening Process

The retrieved records were imported into Zotero 9.0. reference management software, and duplicate records were removed. Two reviewers independently screened the titles and abstracts of all identified studies. Articles that met the inclusion criteria were then assessed in full text.

Full-text screening was conducted independently by multiple reviewers. Any disagreements regarding study eligibility were resolved through discussion within the research team until consensus was reached.

### 2.5. Data Extraction and Synthesis

Relevant information was systematically extracted from each included study using a standardized data-extraction form developed by the research team. The extracted data included author; country and year of publication; study design; participant characteristics; sample size; wearable device type and name; body location of device placement; rehabilitation setting; intervention characteristics; intervention duration; and outcomes related to safety, feasibility, and adherence.

Given the variability in outcome reporting across studies, recruitment rate, retention rate, dropout rate, completion rate, attendance rate, usability, and acceptability were extracted as indicators related to feasibility and participation. Adherence was recorded separately when explicitly reported by the original study authors. Because definitions and measurement approaches varied considerably across studies, no attempt was made to quantitatively pool these outcomes. To improve consistency, recruitment rate was defined as the proportion of eligible individuals who enrolled in a study, completion rate as the proportion of participants who completed the intervention protocol, attendance rate as the proportion of scheduled sessions attended, dropout rate as the proportion of participants who withdrew before study completion, and adherence as the extent to which participants followed the prescribed intervention as defined by the original study authors.

After verification by two reviewers, the extracted data were organized and synthesized using descriptive analysis. Given the substantial heterogeneity in study design, intervention characteristics, wearable device types, rehabilitation settings, and outcome reporting, the findings were summarized narratively rather than quantitatively synthesized.

### 2.6. Critical Appraisal

Consistent with the Joanna Briggs Institute (JBI) methodology for scoping reviews and the PRISMA-ScR guidance, the primary objective of this review was to map and characterize the available evidence rather than to evaluate intervention effectiveness. Therefore, a formal methodological quality assessment or risk-of-bias evaluation was not conducted.

### 2.7. Generative AI Usage Statement

Generative artificial intelligence (GenAI) tools (ChatGPT, GPT-5.5-mini, OpenAI, San Francisco, CA, USA) were used to assist with literature organization, preliminary screening support, and language refinement. However, all study selection decisions were independently performed by two reviewers. Any outputs generated by AI tools were manually verified by the reviewers and were not used as the sole basis for inclusion or exclusion decisions. All data extraction, interpretation of findings, and formulation of conclusions were conducted independently by the research team.

## 3. Results

### 3.1. Results of the Literature Screening

**Figure 1:**
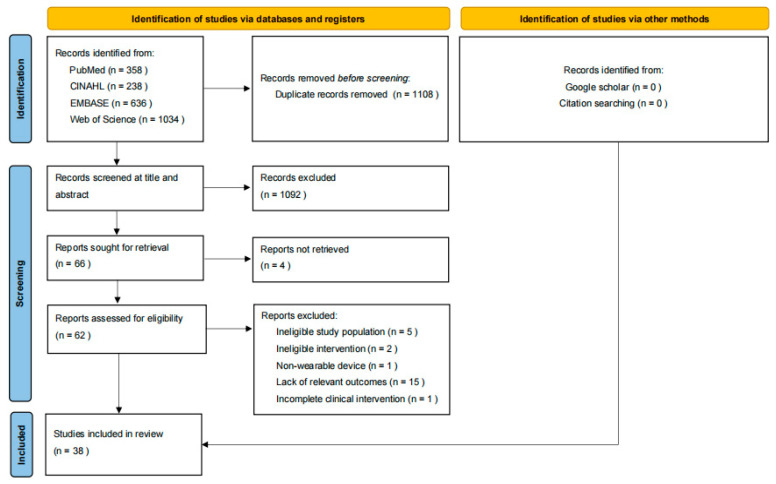
PRISMA diagram on search results.

An initial search of the databases yielded 2266 articles; after removing duplicates, 1158 articles remained. After reviewing the titles, abstracts, and full texts to exclude irrelevant articles, 38 articles were ultimately included. See Figure 1 for the PRISMA flowchart.

### 3.2. Basic Characteristics of Included Studies

The 38 included studies were published between 2020 and 2026 and were conducted across multiple countries, including China, the United States, Canada, South Korea, and Poland, with 12 studies involving international collaborations. Participants represented different stages of stroke, and sample sizes ranged from 5 to 151, with four studies including more than 50 participants. The study designs were primarily randomized controlled trials (15 studies) and feasibility studies (9 studies). The remaining studies included pilot studies (3), qualitative studies (2), and one study each of mixed-methods research, pre–post controlled study, safety study, observational study, prospective comparative study, longitudinal study, multicenter clinical trial, crossover experiment, and wearability assessment study. Although randomized controlled trials constituted the largest study category, a considerable proportion of the included evidence originated from feasibility, pilot, and exploratory studies with relatively small sample sizes. In terms of application settings, 22 studies were conducted in clinical rehabilitation hospitals or outpatient settings, 11 studies were implemented in home environments, and 5 studies were conducted in community or mixed settings. Home-based rehabilitation studies mainly focused on monitoring devices and upper-limb wearable devices, while clinical studies more frequently involved exoskeletons and other devices that require professional assistance.

A wide range of wearable devices were investigated, including exoskeleton systems (e.g., ExoNET and ReWalk ReStore™), wrist-worn devices (e.g., Apple Watch), orthotic devices (e.g., iStride™), inertial motion monitoring devices (e.g., ActiGraph GT9X Link and Fitbit Inspire 2), multimodal monitoring systems (e.g., ARYS™ me|tracker and Przypominajka), pressure or vibrotactile feedback devices (e.g., MusicGlove and Vibrotactile Stimulation Glove), and electrical stimulation devices (e.g., NeuroSkin^®^).

The frequency, duration, and overall period of interventions varied considerably across studies. Individual sessions ranged from 10 to 90 min, with training frequencies of 1–15 sessions per week. Intervention durations ranged from 1 week to 3 months, with most studies lasting 2–8 weeks. Some studies described intervention dosage in terms of the total number of training sessions or cumulative training time, with total sessions ranging from 2 to 60 and weekly training durations from 3 to 21 h. Other studies required participants to wear devices for approximately 10–12 h per day for periods ranging from 1 to 6.4 weeks.

The characteristics of participants in the included studies indicated that wearable devices were mainly used among stroke survivors in the recovery stage, those with mild-to-moderate motor impairment, and individuals aged 60–75 years. In terms of stroke stage, most studies included patients in the recovery phase (approximately 3–6 months post-stroke), followed by those in the acute or subacute phase, while patients in the chronic phase were less frequently represented.

For the purpose of evidence mapping, wearable devices were categorized according to their primary rehabilitation function and mechanism of action. Based on the predominant role of the device during rehabilitation, devices were classified as mechanically assisted wearable devices, wearable systems for movement monitoring and biofeedback, or wearable devices based on electrical or sensory stimulation. Mechanically assisted devices, such as exoskeletons or soft robotic systems, were most commonly used for gait and upper-limb function training but generally involved greater operational complexity and wearing burden. In contrast, monitoring and feedback devices were more often used for activity assessment or home-based rehabilitation monitoring and demonstrated better scalability. Stimulation-based devices were less frequently studied but showed potential in enhancing sensory input and neuromodulation. These technological approaches reflect the diverse functional roles and application scenarios of wearable rehabilitation devices. Due to substantial discrepancies in study design, intervention protocols, outcome measures and rehabilitation settings, direct horizontal comparisons between different categories of devices are difficult to conduct (see Table 1).

To improve the readability of the study characteristics, supplementary summary tables were created to present the distribution of included studies according to device category (Table S1), stroke stage (Table S2), and rehabilitation setting (Table S3).

### 3.3. Safety

Among the 38 included studies, adverse events occurring during the intervention period were reported in varying degrees of detail. Most studies explicitly reported no serious device-related adverse events, although considerable variation was observed in adverse event monitoring and reporting methods across studies. Reported adverse events were predominantly mild and included skin redness or pressure marks, muscle fatigue, localized pain, and transient discomfort. These events most commonly occurred during the initial wearing stage or following increases in training intensity and were largely manageable through device adjustment, parameter modification, or adaptation of the training protocol.

Differences in adverse events were observed across device types. Upper-limb systems (e.g., wrist exoskeletons, soft robotic gloves, and upper-limb support devices) rarely reported serious adverse events; when they occurred, they were mainly muscle fatigue, mild pain, or skin redness, which could usually be relieved through adjustments. In contrast, adverse events associated with lower-limb walking devices were relatively more complex. In addition to fatigue, knee pain, skin abrasions, erythema, blisters, or bruising were reported. One study documented participant withdrawal due to knee pain and fatigue [23]. In the Atalante hands-free exoskeleton study, two serious adverse events (dizziness and dysarthria) were reported but were determined to be unrelated to the device; however, knee pain and skin injuries were observed, and three device-related withdrawals occurred, suggesting that skin pressure and joint loading issues are relatively common in lower-limb systems [33].

In addition, studies of home-based gait devices reported cases of “controlled falls” and withdrawal due to dizziness [42]. Although these events did not result in serious injuries, they highlight the importance of balance assessment and close supervision during initial use in non-clinical environments. Wrist-worn or monitoring devices reported fewer adverse events, which were mainly mild skin discomfort or inconvenience during wearing.

Overall, reported adverse events can be broadly categorized into mechanical or contact-related issues, load-related problems, and environmental or system-related risks. Although current evidence suggests that wearable devices are generally well tolerated and that serious device-related adverse events are uncommon, interpretation of safety findings should be undertaken with caution. Variability in adverse event definitions, monitoring procedures, reporting standards, and participant populations across studies limits direct comparison and precludes reliable estimation of the true frequency of device-related adverse events.

### 3.4. Feasibility

#### 3.4.1. Recruitment Feasibility

Recruitment outcomes varied substantially across studies. Reported recruitment rates ranged from 5.8% to 100%, indicating considerable variability in participant eligibility, willingness to participate, and implementation requirements.

Several studies achieved relatively high recruitment rates, including a portable wrist exoskeleton study (100%) [27], a wearable activity monitor study (90.74%) [50], and a qualitative study of exoskeleton-based physiotherapy (87.5%) [26]. However, recruitment was substantially lower in studies with more restrictive eligibility criteria or greater participant demands. For example, a soft robotic hand orthosis study enrolled only 11 participants from 71 screened individuals (16%) [33], while another study recruited 41 participants from 707 screened cases (5.8%) [59]. Studies requiring higher levels of cognitive ability, independent technology use, caregiver support, or severe impairment management generally experienced greater recruitment challenges.

Overall, these findings suggest that recruitment may represent a significant implementation barrier for some wearable rehabilitation programs, particularly those involving complex devices, intensive training requirements, or highly selected patient populations.

#### 3.4.2. Implementation Feasibility

Most studies reported that wearable devices could be successfully implemented in stroke rehabilitation settings; however, the level of support required varied considerably across device types and rehabilitation environments.

Exoskeletons and robotic systems typically required assistance from physical therapists for device fitting, parameter adjustment, and safety monitoring. Several studies reported lengthy setup procedures, the need for individualized calibration, and substantial therapist involvement during training sessions [26,31,35]. Qualitative evidence further suggested that device preparation time, operational complexity, and user comfort could influence implementation efficiency and user acceptance.

Upper-limb wearable devices were generally easier to operate than lower-limb exoskeleton systems. Nevertheless, many studies reported that participants required a familiarization period before independent use could be achieved [33]. In home-based rehabilitation studies, participants often became more independent over time, reducing the need for caregiver or therapist assistance [43]. However, several home-based interventions still required caregiver supervision, technical support, or regular follow-up visits to ensure safe and effective use [45,54].

These findings suggest that implementation feasibility is influenced not only by device design but also by the level of professional support, participant characteristics, and rehabilitation setting.

#### 3.4.3. Technical Feasibility

Most studies reported that wearable devices were capable of supporting rehabilitation activities and generally demonstrated acceptable technical performance. Commonly reported strengths included stable sensing performance, adequate battery life, reliable data collection, and satisfactory usability ratings.

Several studies reported favorable usability outcomes, with System Usability Scale (SUS) scores ranging from 72.5 to 85.4 [33,53,59]. Multicenter studies further demonstrated that wearable technologies could be deployed across different rehabilitation sites with acceptable operational performance [29,58].

Nevertheless, technical challenges were frequently reported. These included mechanical component failures, sensor malfunction, battery limitations, software lag, delayed system responses, Bluetooth connectivity issues, and data loss [33,37,52,56,60]. One study reported a device failure rate of 11.6% before software upgrades, which decreased to 3.4% after system optimization [37]. Another study found that only 87.6% of collected monitoring data were considered valid because of sensor-related issues [46].

Overall, current evidence suggests that wearable technologies are technically feasible for stroke rehabilitation. However, device fit, system stability, battery performance, data reliability, and ease of operation remain important areas for further improvement.

### 3.5. Participation- and Adherence-Related Outcomes

#### 3.5.1. Adherence

In this review, adherence was defined as the extent to which participants followed the prescribed intervention protocol, such as completing the recommended training dosage, wearing duration, or intervention schedule. Only four studies explicitly reported adherence-related outcomes, and the definitions and measurement approaches varied considerably across studies, limiting comparability.

Among the available studies, adherence appeared to be influenced by multiple factors, including device comfort, ease of use, training burden, technical support, and participants’ self-management abilities. Home-based interventions appeared particularly sensitive to factors such as technological familiarity, environmental support, and the ability to independently complete prescribed training tasks. Due to the limited number of studies and inconsistent reporting methods, no clear conclusions regarding adherence across different device types or rehabilitation settings could be established.

#### 3.5.2. Completion and Attendance

Several studies reported high intervention completion rates under supervised clinical conditions. Completion rates were close to or reached 100% in studies involving the Kickstart lower-limb exoskeleton [24], EAMT (Exoskeleton-Assisted Anthropomorphic Movement Training) upper-limb training [25], and a feasibility study of a portable wrist exoskeleton [27]. Other studies similarly reported high completion rates within structured rehabilitation settings [35,36,39,41,46].

Attendance outcomes were reported less frequently. One home-based gait-training study reported an attendance rate of approximately 97.5% and a completion rate of approximately 91.3% [42]. Regarding training dosage, most lower-limb training protocols involved 20–60 min per session, whereas upper-limb training typically lasted 30–45 min per session, with a frequency of three to five sessions per week. These training intensities were generally well tolerated by participants. Some studies adopted longer wearable interventions, such as wearing upper-limb devices for approximately five hours per day [41]. Under supervised clinical conditions, these training dosages were often associated with high completion rates.

These findings suggest that wearable device interventions are generally acceptable to participants within structured rehabilitation programs. However, completion and attendance rates reflect engagement in study procedures and should not be interpreted as direct measures of adherence to prescribed rehabilitation interventions.

#### 3.5.3. Dropout and Withdrawal

Several studies reported participant dropout or withdrawal during the intervention period. A multicenter randomized controlled trial of lower-limb exoskeleton gait training reported a dropout rate of 21.6% in the intervention group, primarily due to fatigue or participants’ requests to discontinue training [29]. Similarly, a study of the ReStore soft exosuit reported a completion rate of approximately 81.8%, suggesting that factors such as wearing burden, training fatigue, and participant motivation may influence continued participation [36].

In home-based rehabilitation studies, dropout rates appeared more variable. A home-based training study using a music-based game reported an overall completion rate of only 46% [55], whereas another study reported a dropout rate of approximately 36% despite relatively high Intrinsic Motivation Inventory (IMI) scores [56]. One study excluded approximately 45% of participants during data analysis because of insufficient adherence or inadequate data quality [51], while another reported that approximately 22% of participants withdrew because they were unfamiliar with information technology devices [49].

Qualitative evidence further suggested that prolonged setup procedures, insufficient device comfort, technical difficulties, and increased operational burden for both patients and therapists may negatively affect continued participation [30]. These barriers appeared particularly relevant in home-based interventions and in devices requiring greater physical effort or technical support.

Overall, adherence related evidence remains limited. Most studies reported completion, attendance, or dropout outcomes rather than adherence itself, and substantial variability existed in the definitions and measurement methods used across studies. Consequently, the available evidence is insufficient to draw firm conclusions regarding adherence to wearable device interventions in stroke rehabilitation.

## 4. Discussion

### 4.1. Low Recruitment Rates: Mismatch Between Device Characteristics and Functional Status of Stroke Populations

This review found that some studies on wearable rehabilitation devices reported relatively low recruitment rates or high screening exclusion ratios. This phenomenon is not only related to study design or sampling sources but also reflects a mismatch between device characteristics and the functional status of stroke patients.

Many wearable devices emphasize active participation and human–machine interaction. While this approach may promote neuroplasticity and improve training specificity, it also requires patients to have certain levels of motor ability, cognitive function, and task execution capacity. For example, upper-limb devices often require active triggering or task-based feedback, while lower-limb exoskeleton systems typically require sufficient trunk control and balance. In contrast, traditional passive therapies place fewer demands on patient participation and are therefore applicable to a broader population. As device interactivity increases, the functional threshold for participation may also rise, potentially limiting the proportion of eligible patients. The complexity of device operation and human–machine interaction has also been identified as a potential barrier to technology adoption among older adults and individuals with neurological impairments [61].

Technical barriers associated with digital operation may also act as implicit screening factors. Many devices rely on mobile terminals, Bluetooth connections, or cloud-based data synchronization. Previous studies have indicated that age, cognitive level, and prior experience with technology may influence patients’ ability to use such systems independently. For instance, one study reported [35] that an 84-year-old participant withdrew from the study due to difficulty learning to use the rehabilitation technology, while other participants required extensive guidance despite normal cognitive function. These findings suggest that digital self-efficacy may influence participation willingness even when devices are designed to be user-friendly. Similar findings have been reported in previous rehabilitation technology studies, in which participants expressed reluctance to adopt unfamiliar technologies or withdrew because of difficulties learning device operation [62].

Furthermore, wearable devices often require prolonged or frequent use, which may impose additional time demands or physical and psychological burdens. For stroke survivors already coping with functional impairments, these factors may further reduce willingness to participate.

Therefore, low recruitment rates may reflect a structural mismatch between highly interactive, technology-dependent devices and the diverse functional capacities of stroke patients. Future research should consider reducing dependence on residual motor function and technical skills by optimizing user interfaces, shortening learning curves, and improving wearing comfort. Incorporating digital health literacy assessments and providing tiered training may also help reduce technology-related barriers and broaden the potential user population.

### 4.2. Safety Considerations Across Different Device Types

This review identified notable differences in safety profiles between upper-limb and lower-limb wearable devices. Upper-limb systems, including wrist exoskeletons, soft robotic gloves, and arm-support devices, were generally associated with relatively few adverse events. When adverse events occurred, they were typically mild and transient, such as muscle fatigue, localized discomfort, or temporary skin irritation. These issues were often resolved through adjustments to device fit, training intensity, or wearing duration. In contrast, lower-limb wearable devices, particularly exoskeletons and exosuits designed for gait rehabilitation, appeared to be associated with a broader range of adverse events. In addition to fatigue, studies reported knee pain, skin abrasions, erythema, bruising, and occasional participant withdrawal due to discomfort. Although serious device-related adverse events were rarely reported, lower-limb systems place greater mechanical demands on users because they directly interact with weight-bearing joints and locomotor functions.

Several factors may contribute to these differences. Compared with upper-limb devices, lower-limb systems generally require longer wearing times, more complex fitting procedures, and greater physical effort during use. Furthermore, stroke-related impairments such as muscle weakness, abnormal gait patterns, joint contractures, and lower-limb spasticity may affect device fit and pressure distribution, thereby increasing the risk of discomfort and skin-related complications. Spastic equinovarus patterns involving the gastrocnemius, soleus, and tibialis muscles may be particularly relevant because they can influence lower-limb alignment and device compatibility. Recent evidence suggests that targeted spasticity management, including ultrasound-guided botulinum toxin injections, may improve limb positioning and potentially enhance device tolerance and wearability [63].

These findings have important implications for clinical implementation. For lower-limb wearable systems, careful patient selection, individualized device fitting, routine skin inspection, and gradual progression of training intensity may help reduce adverse events. Additional attention may be required for patients with severe spasticity, joint deformities, or substantial balance impairments. In contrast, upper-limb devices may be easier to integrate into routine rehabilitation programs because of their lower physical burden and simpler operational requirements. Nevertheless, regular monitoring of comfort and fatigue remains important across all wearable device categories.

### 4.3. From Clinical to Home-Based Rehabilitation: Challenges in Real-World Implementation

With the development of telemedicine and digital health, an increasing number of studies have begun to explore the application of wearable devices in home-based rehabilitation. However, compared with controlled clinical environments, home settings present greater uncertainty in terms of safety management, technical support, and patients’ self-management abilities.

In clinical settings, device use is typically supervised by physical therapists, allowing patients to complete device donning, parameter adjustments, and training tasks under professional guidance. In home environments, however, professional support is reduced, making patients’ self-management abilities and device usability critical factors. In addition, variations in home space layout, floor conditions, and safety supervision may increase potential risks. For example, one study reported that training was interrupted due to interference from a pet, highlighting the need for attention to safety management in home settings [42].

Device operational complexity is also an important factor limiting home-based adoption. Some exoskeleton systems require considerable time for donning and parameter setup, and without therapist assistance, such complex procedures may increase user burden and reduce the likelihood of sustained use. Furthermore, ergonomic issues, such as unnatural gait patterns or discomfort during wear, may negatively affect the user experience.

Adherence to wearable device interventions in home environments also shows considerable individual variability. Patients’ emotional status, daily routines, and familiarity with technology may all influence the frequency of device use. For stroke survivors, emotional distress or reduced self-efficacy during the early recovery stage may further affect long-term training adherence.

Moreover, most existing studies have focused on short-term interventions, and evidence regarding the long-term effectiveness and safety of home-based use remains limited. Factors such as changes in daily routines, fluctuations in health status, and differences in family support may influence the sustainability and effectiveness of device-based interventions. Therefore, future research should include longer follow-up periods to evaluate the long-term effectiveness and sustainability of wearable devices in home-based rehabilitation.

The transition of wearable devices from clinical to home-based rehabilitation involves not only technological challenges but also factors related to ergonomics, patient behavior, home environments, and telemedicine support. Future device design should place greater emphasis on user experience, such as simplifying the donning process, optimizing ergonomic design, and improving system stability. In addition, integrating remote monitoring and technical support platforms may provide patients with continuous guidance, thereby improving the feasibility and long-term adherence of home-based rehabilitation. Future implementation research should also examine whether safety, feasibility, and adherence differ systematically according to device type, rehabilitation setting, and stroke severity, as current evidence remains insufficient to support definitive conclusions.

### 4.4. Limitations

This review has several limitations. First, as a scoping review, the aim of this study was to systematically map the characteristics and trends of existing evidence rather than to conduct a rigorous quality assessment or risk-of-bias evaluation of the included studies. Therefore, the findings mainly provide an overview of the current state of research rather than a quantitative evaluation of intervention effectiveness.

Second, the search was restricted to studies published between 2020 and 2026, reflecting the period of rapid advancement in wearable rehabilitation technology. While this temporal scope ensures relevance to current clinical practice, it may have excluded earlier foundational work. Furthermore, the inclusion of only English- and Chinese-language literature may have introduced language bias, potentially limiting the generalizability of findings across other research communities.

Third, considerable heterogeneity existed among the included studies in terms of device types, intervention modes, study designs, and follow-up durations, which limited the direct comparability of results across studies. The reporting of participant characteristics varied considerably across studies, with many failing to provide complete information on age, gender distribution, or severity of motor impairment. This inconsistency limited the ability to draw conclusions about which patient populations may benefit most from wearable device interventions, and future studies should adopt more standardized reporting of participant demographics. Due to the descriptive nature of this review and the heterogeneity of the available evidence, we were unable to determine whether safety, feasibility, or adherence differed systematically according to device type, rehabilitation setting, or stroke severity. Future studies using more standardized reporting frameworks may facilitate such comparisons.

Fourth, adverse event reporting was inconsistent across studies, and only a limited number of studies provided detailed descriptions of adverse event monitoring procedures. Variations in adverse event definitions, surveillance methods, and reporting practices limited comparability across studies and made it difficult to accurately characterize the overall safety profile of wearable devices. Moreover, the absence of reported adverse events in some studies should not be interpreted as evidence that wearable devices are inherently safe, as underreporting or inconsistent reporting practices may have contributed to the apparent absence of reported adverse events.

Fifth, adherence data were particularly sparse. Given this limited and inconsistent evidence base, conclusions regarding adherence should be interpreted with caution, and future research should adopt standardized, operationally defined adherence metrics.

Finally, many included studies were pilot or feasibility studies with relatively small sample sizes and primarily focused on short-term interventions. These characteristics may limit the external validity and generalizability of the current findings. Evidence regarding the long-term safety, adherence, and effectiveness of wearable devices in stroke rehabilitation remains limited. Therefore, future large-scale pragmatic implementation studies with longer follow-up periods are needed to further validate the potential value of wearable devices in stroke rehabilitation.

## 5. Conclusions

This scoping review provides an overview of current evidence regarding wearable devices in stroke rehabilitation. Existing studies suggest that wearable technologies may be implemented across a range of rehabilitation settings, although substantial heterogeneity exists in the reporting of safety and feasibility outcomes. Most reported adverse events were mild and manageable; however, variations in adverse event monitoring and reporting methods make it difficult to determine the true incidence of device-related complications.

Evidence regarding adherence remains limited because only a small number of studies explicitly reported adherence outcomes, and substantial variation existed in the definitions and measurement methods used. Consequently, conclusions regarding adherence should be interpreted cautiously.

Wearable devices show promise for supporting motor recovery, increasing rehabilitation intensity, and facilitating rehabilitation beyond traditional clinical settings. Nevertheless, challenges related to device usability, participant recruitment, therapist support requirements, and long-term implementation remain. These findings should be interpreted in light of the substantial heterogeneity of the included studies, the limited availability of adherence data, and the absence of formal methodological quality appraisal.

Future research should adopt standardized reporting frameworks for safety, feasibility, and adherence outcomes, while conducting larger and longer-term studies to better understand the real-world implementation and sustainability of wearable technologies in stroke rehabilitation.

## Figures and Tables

**Table 1:** Basic characteristics of included studies on wearable rehabilitation devices.

Authors	Country	Study Design	Sample	Device	Setting
Louie [23]	Canada	RCT *	*n* = 17 control *n* = 19 stroke, subacute	EksoGT	Clinical
Liang [24]	China	RCT	*n* = 23 control *n* = 23 stroke, subacute	Kickstart^®^ Walk Assist	Clinical
Chen [25]	China	RCT	*n* = 10 control *n* = 10 stroke, subacute	EAMT upper limb exoskeleton	Clinical
Louie [26]	Canada	Qualitative descriptive study	*n* = 20, subacute	EksoGT	Clinical
Lambelet [27]	Switzerland	Wearability evaluation & device characterization	*n* = 2 stroke, chronic *n* = 15 healthy	eWrist	Clinical & Home
Ambrosini [28]	Italy, Austria, Germany, etc.	RCT	*n* = 36 control *n* = 36 stroke, subacute	RETRAINER	Clinical
Chang [29]	South Korea, Malaysia	RCT	*n* = 77 control *n* = 74 stroke, subacute	ANGEL LEGS M20	Clinical
Vaughan-Graham [30]	Canada	Qualitative study	*n* = 5 stroke, chronic *n* = 6 therapists	H2 exoskeleton-assisted	Clinical
HSU [31]	Taiwan, China	Single-arm feasibility study	*n* = 12 stroke, chronic	HS 001	Clinical
Tanczak [32]	Singapore, Switzerland	Two-phase feasibility study	*n* = 8 stroke, chronic	RELab Tenoexo 2.0	Clinical & Home
Lejeune [33]	France, Belgium, Luxembourg	Prospective multicenter safety study	*n* = 40 stroke (subacute & chronic)	Atalante	Clinical
Yao [34]	China, USA	Single-arm pre–post feasibility study	*n* = 30 stroke (subacute & chronic)	Kickstart^®^ Walk Assist	Clinical
E Proulx [35]	Canada	Prospective non-randomized controlled study	*n* = 5 control *n* = 6 stroke	Dexmo glove	Clinical
Awad [36]	USA	Multicenter clinical trial	*n* = 44 stroke (>2 weeks post-stroke)	ReWalk ReStore™	Clinical
Macaluso [37]	USA, South Korea	Prospective single-arm longitudinal study	*n* = 41 stroke (subacute & chronic)	GEMS-H	Clinical
Doronzio [38]	Italy, Switzerland	Non-randomized pilot study	*n* = 10 stroke, chronic	Myosuit	Clinical
Noronha [39]	Singapore, Switzerland, Germany, Belgium	Feasibility single-centre open label clinical trial	*n* = 10 stroke, (subacute & chronic)	Exoskeleton-assisted	Clinical
Celian [40]	USA	RCT	*n* = 10 control *n* = 9 stroke, chronic	ExoNET	Clinical
Xu [41]	China	RCT	*n* = 23 control *n* = 25 stroke, subacute	Wearable hand orthosis	Clinical
Huizenga [42]	USA	Single-arm pre–post study	*n* = 21 stroke, chronic	iStride™	Home
Seo [43]	USA	Feasibility study	*n* = 19 stroke (acute & subacute)	ActiGraph GT9X Link	Home
Darcy [44]	USA	Non-randomized pilot feasibility study	*n* = 5 stroke, chronic	iStride	Home
Demers [45]	USA	Observational study	*n* = 30 stroke, chronic	MiGo system	Home & Community
Lu [46]	China	RCT	*n* = 34 control *n* = 45 stroke, subacute	Stroke Intelligent Rehabilitation Training System	Clinical
Marek [47]	Poland	RCT	*n* = 8 control *n* = 8 stroke, (subacute & chronic)	Przypominajka	Clinical & Home
Tse [48]	Australia	Single-group pre-post pilot study	*n* = 12 stroke, subacute	ActiGraph wGT3X-BT/GT9X Link	Clinical
Chae [49]	South Korea	Prospective comparative study	*n* = 6 control *n* = 17 stroke, chronic	LG W270 smartwatch	Home
Nam [50]	USA	Feasibility study	*n* = 65 stroke, chronic	Fitbit Inspire 2	Home
Mayrhuber [51]	Singapore, Switzerland, USA	RCT	*n* = 23 control *n* = 19 stroke, chronic	ARYS™ me|tracker	Home
Langerak [52]	Netherlands	Study Design Crossover study	*n* = 17 stroke, subacute	Arm Activity Tracker	Clinical
Toh [53]	Hong Kong, China, Singapore	RCT	*n* = 6 control *n* = 6 stroke, chronic	Smart Reminder	Home
Toh [54]	Hong Kong, China, Singapore	Mixed-methods study	*n* = 11 stroke, chronic	Smart Reminder	Home
Sanders [55]	USA	RCT	*n* = 5 control *n* = 6 stroke, subacute	MusicGlove	Home
Hung [56]	USA	RCT	*n* = 8 control *n* = 15 stroke, chronic	MINT *	Home
Metani [57]	France, Serbia, Luxembourg, Italy	Multicenter retrospective feasibility study	*n* = 15 stroke, subacute *n* = 7 therapists	NeuroSkin^®^	Clinical
Egger [58]	Switzerland	RCT	*n* = 21 control *n* = 20 stroke, subacute	RSS + ArmeoPower	Clinical
Seim [59]	USA	RCT	*n* = 8 control *n* = 8 stroke, chronic	Vibrotactile Stimulation Glove	Home
Wang [60]	USA	Pilot study	*n* = 5 stroke, chronic *n* = 5 therapists	FoVi	Home & Community

* RCT: Randomized controlled trial; MINT: Myoelectric Interface Neurorehabilitation training.

## Data Availability

No new data were created or analyzed in this study. Data sharing is not applicable to this article.

## Supplementary Materials

The following supporting information can be downloaded at: https://www.mdpi.com/article/10.3390/healthcare14152400/s1; File S1: Search Strategy; Table S1: Distribution of included studies by wearable device category; Table S2: Distribution of included studies by stroke stage; Table S3: Distribution of included studies by rehabilitation setting.

## Abbreviations

The following abbreviations are used in this manuscript:

PRISMA-ScR	Preferred Reporting Items for Systematic Reviews and Meta-Analyses Extension for Scoping Reviews
RCT	Randomized controlled trial
MINT	Myoelectric Interface Neurorehabilitation training
IMI	Intrinsic Motivation Inventory
