# Supplementary material for: Clinical Application of Wearable Devices in Stroke Rehabilitation: A Scoping Review of Safety, Feasibility, and Adherence
Source: Healthcare (Basel). 2026 Aug 5;14(15):2400. doi: 10.3390/healthcare14152400 (PMC13465213; doi:10.3390/healthcare14152400)
Supplement: Supplementary file 1 [file healthcare-14-02400-s001.zip › Search Strategy.pdf]

Search Strategy		
Database	Search query	2020-2026
PubMed	(("Stroke"[Mesh] OR stroke*[Title/Abstract] OR "cerebrovascular accident*"[Title/Abstract] OR poststroke[Title/Abstract] OR hemiplegia[Title/Abstract] OR hemiparesis[Title/Abstract]) AND("Wearable Electronic Devices"[Mesh] OR "Exoskeletons"[Mesh] OR wearable*[Title/Abstract] OR "smart watch*"[Title/Abstract] OR exoskeleton*[Title/Abstract] OR "activity monitor*"[Title/Abstract] OR "motion sensor*"[Title/Abstract] OR "inertial measurement unit*"[Title/Abstract] OR accelerometer*[Title/Abstract]) AND("Feasibility Studies"[Mesh] OR "Patient Satisfaction"[Mesh] OR safety[Title/Abstract] OR "adverse event*"[Title/Abstract] OR feasibility[Title/Abstract] OR adherence[Title/Abstract] OR compliance[Title/Abstract] OR usability[Title/Abstract] OR acceptability[Title/Abstract]))NOT Review[Publication Type]	358
CINAHL	(MH "Stroke" OR MH "Stroke Patients" MH "Ischemic Stroke" OR MH "Hemorrhagic Stroke") AND (MH "Wearable Sensors/AE" OR MH "Exoskeleton Devices" OR MH "Assistive Technology Devices" MH "Exoskeleton Devices" OR MH "Robotics")	238
Embase	(('stroke'/exp OR stroke*:ti,ab OR 'cerebrovascular accident*':ti,ab OR poststroke:ti,ab OR hemiplegia:ti,ab OR hemiparesis:ti,ab) AND('wearable electronic device'/exp OR 'exoskeleton'/exp OR wearable*:ti,ab OR smartwatch*:ti,ab OR exoskeleton*:ti,ab OR 'activity monitor*':ti,ab OR 'motion sensor*':ti,ab OR 'inertial measurement unit*':ti,ab OR accelerometer*:ti,ab) AND('feasibility study'/exp OR 'patient satisfaction'/exp OR safety:ti,ab OR 'adverse event*':ti,ab	636

	OR feasibility:ti,ab OR adherence:ti,ab<br>OR compliance:ti,ab OR usability:ti,ab<br>OR acceptability:ti,ab))NOT ('review'/it OR 'systematic review'/it OR 'meta analysis'/it)	
Web of Science	TS=((stroke* OR "cerebrovascular accident*" OR poststroke OR hemiplegia OR hemiparesis)<br>AND(wearable* OR "wearable device*" OR smartwatch* OR exoskeleton* OR "activity monitor*" OR "motion sensor*"<br>OR "inertial measurement unit*" OR accelerometer*)AND(safety OR "adverse event*" OR feasibility OR adherence<br>OR compliance OR usability OR acceptability))NOT TS=("systematic review" OR "meta-analysis" OR "scoping review")	1034
