# Supplementary material for: Clinical Application of Wearable Devices in Stroke Rehabilitation: A Scoping Review of Safety, Feasibility, and Adherence
Source: Healthcare (Basel). 2026 Aug 5;14(15):2400. doi: 10.3390/healthcare14152400 (PMC13465213; doi:10.3390/healthcare14152400)
Supplement: Supplementary file 1 [file healthcare-14-02400-s001.zip › Supplementary tables.pdf]

Supplementary Table S1 Distribution of included studies by wearable device category

Device category	Number of studies
Mechanically assisted wearable devices	20
Wearable systems for movement monitoring	14
Biofeedback/wearable devices based on electrical or sensory stimulation	4

Supplementary Table S2 Distribution of included studies by stroke stage

Stroke stage	Number of studies
Acute & Subacute	1
Subacute	13
Chronic	17
Subacute & Chronic	5
Not reported	2

Supplementary Table 3 Distribution of included studies by rehabilitation setting

Setting	Number of studies
Clinical	22
Home	11
Clinical & Home	3
Home & Community	2
